# A retrospective study on the effect of Chinese patent medicine combined with conventional treatment on the survival outcomes of 313 patients with stage II–III NSCLC

**DOI:** 10.18632/aging.205697

**Published:** 2024-03-28

**Authors:** Kegang Jia, Chenxu Zhang, Fang Li, Bin He, Shenglong Xie, Jun Du, Gang Feng

**Affiliations:** 1 Department of Thoracic Surgery, Sichuan Provincial People’s Hospital, School of Medicine, University of Electronic Science and Technology of China, Chengdu, China; 2 Chengdu Diao Pharmaceutical Group Co., Ltd., Chengdu, China

**Keywords:** non-small cell lung cancer, Chinese patent medicine, Huisheng oral solution, blood hypercoagulable state, survival rate

## Abstract

Purpose: We aim to explore the effect of Chinese Patent Medicine (CPM), including Huisheng oral solution (HSOS) on the 4-year survival rate of patients with stage II and III non-small cell lung cancer, and assess the association between blood coagulation indicators and survival outcomes.

Materials and Methods: 313 patients diagnosed with stage II and III NSCLC were collected during 2015-2016. Kaplan-Meier method and Cox proportional hazard model were applied to analyze the factors affecting the 4-year survival rate of patients.

Results: According to the effect of CPM, the medicine prescribed in this study could be classified into two types. The proportion of patients who received “Fuzheng Quyu” CPM for more than three months was higher than the proportion of patients who received other two types of CPM for more than three months. Medical records of 313 patients with NSCLC were analyzed. 4-year survival rate for patients received CPM more than 6 months and 3 months were higher than those received CPM less than 3 months (*P* = 0.028 and *P* = 0.021 respectively. In addition, 4-year survival rate for patients who received HSOS for more than 3 months was higher than those who received HSOS for less than 3 months (*P* = 0.041). Patients with elevated preoperative fibrinogen (FIB) level and those without surgery had an increased mortality risk (HR = 1.98, *P* < 0.01, and HR = 2.76, *P* < 0.01 respectively).

Conclusion: The medium and long-term use of CPM/HSOS was positively associated with higher survival rate in NSCLC patients. Patients with high-level preoperative FIB level and those without surgery might have a poor prognosis in the following years.

## INTRODUCTION

Lung cancer is the leading cause of cancer death [1]. Approximately 80% to 85% of lung cancer cases were NSCLC [2]. In 2020, nearly 2.20 million people were diagnosed with lung cancer, and approximately 1.80 million people died from lung cancer [1]. The high mortality of lung cancer is closely related to its biological characteristics of easy metastasis and recurrence. Sugimura and co-workers found that more than 40% NSCLC patients who underwent complete resection experienced cancer recurrence and the median disease-free survival (DFS) was 11.5 months, while the median survival time was 8.1 months after the recurrence of tumor [3]. In terms of the mechanism of tumor recurrence and metastasis, some research believe that the blood hypercoagulable state is closely related to immune escape, adhesion and metastasis of tumor cells [4–6]. Choi PJ and Farge D reported that elevated preoperative platelet (PLT) levels increase the risk of NSCLC progression [7, 8]. Lin Y and Can Hou indicated that D-dimer and fibrinogen (FIB) may play important roles in predicting the tumor prognosis [9, 10], and patients with at least two of three coagulation indicators elevated (D-dimer, FIB and PLT) have a significantly higher risk of cancer progression than those of patients with one coagulation indicator elevated.

There are many syndrome types in stage II–III NSCLC patients, such as blood stasis, Qi deficiency, Qi stagnation, toxin retention, generation of dampness, and so on [11–13]. Above findings that hypercoagulable state increases the risk of tumor recurrence and metastasis are highly consistent with the Traditional Chinese Medicine (TCM) theory that blood stasis syndrome promotes tumor recurrence and metastasis. Therefore, as the crystallization of TCM theory, varieties of Chinese patent medicine (CPM) such as heat-clearing and detoxicating medicine, medicine of promoting blood circulation and removing blood stasis, and medicine of nourishing healthy qi and eliminating blood stasis are widely used to treat the tumor. However, it is challenging to assess the efficacy of most anti-tumor Chinese medicine, which largely attributed to the lack of rigorous clinical research design and the characteristics of complex component, multi-target, and low-medium action intensity of TCM. In addition, most of the previous studies ignore the effective exposure dose (cumulative treatment duration) of TCM, which somewhat limits the further development of CPM. Encouragingly, several researchers have focused on these issues by conducting rigorous prospective cohort studies. The results have shown that the more extended use of TCM, the higher survival rate of patients with stage II and III colorectal cancer would be [14], and long-term use of TCM was a protective factor for preventing the recurrence and metastasis of NSCLC [15].

Huisheng oral solution, a CPM composed of 34 Chinese medicine components, is commonly prescribed to treat tumor in China. Previous experiments demonstrated that HSOS exerted anti-tumor effect via enhancing body immunity, promoting blood circulation and removing blood stasis [16–19] and reduce the level of PLT and FIB in SD rats [20]. Another animal experiment found that HSOS showed benefits in reducing the level of FIB, down-regulating the expression of related factors in C57 mice, and improving blood hypercoagulative state, thus achieved anti-tumor effects [21]. However, few epidemiologic studies have focused on the association between different cumulative treatment durations of CPM/HSOS and NSCLC patient’s survival rate. Therefore, this study was conducted to evaluate the effect of CPM/HSOS on four-year survival rate of patients with stage II and III NSCLC, and to figure out the relationship between blood coagulation indicators and survival outcomes.

## MATERIALS AND METHODS

### Participants

1301 patients were diagnosed with lung cancer in Sichuan Provincial People’s Hospital during 2015–2016. 313 patients among them were finally included in statistical analysis according to the inclusion and exclusion criteria. The follow-up period was 48 months, 170 (54.3%) patients died in 4 years, 43 (13.7%) patients lost to follow-up, and 100 (31.9%) patients were still alive until the end of this study. The study was reviewed and approved by the Ethics Committee of Sichuan Provincial People’s Hospital (Number: 2019-205). Written informed consent for participation was acquired.

### Inclusion criteria

Patients included in the study had to meet the following items: (1) Patients diagnosed with stage II and III NSCLC (2015 NCCN Guidelines: Non-Small Cell Lung Cancer, [22]) in Sichuan Provincial People’s Hospital during 2015–2016. (2) Patients who had a proper compliance of the study with no less than 2 records of return visit.

### Exclusion criteria

The patients were manually excluded if any of the following items were met: (1) Patients not meeting the inclusion criteria; (2) Patients with other solid tumor or hematological tumor; (3) Patients with a history of thrombosis, or complicated with severe cardiovascular and cerebrovascular diseases, severe coagulation dysfunction, and severe rheumatic diseases; (4) Patients with other conditions deemed unsuitable for inclusion by the researchers; (5) Patients who had serious complications (heart failure, respiratory failure and deep coma not caused by venous thrombus embolism) or died within 30 days after enrollment or surgery; (6) Patients with uncontrollable neurological, psychiatric or mental disorders; (7) Patients with no follow-up record after admission.

### Groups

(1) To compare the survival outcomes between the patients who received CPM for less than 3 months and those who received CPM for more than 3 months; (2) To compare the survival outcomes between the patients who received CPM for less than 3 months and those who received CPM for more than 6 months; (3) To compare the survival outcomes between the patients who received HSOS less than 3 months and those who received HSOS more than 3 months. The treatment duration was defined as the cumulative days of receiving such treatment recorded in the Hospital Information System (HIS) during the study period.

### Follow-up

The follow-up period was 48 months (4 years), and there were 7 data collection points and 1 final telephone follow-up point. All kinds of examination, medication records and outcome of each patient were collected at the time of enrollment, and 1 month, 3 months, 6 months, 1 year, 2 years, and 4 years after enrollment respectively.

### Outcomes

The primary endpoint was death caused by NSCLC in 4 years. Blood routine and preoperative coagulation indicators were also collected at each observation point.

### Statistical analysis

The number of cases, mean, standard deviation, *t*-value and F value of continuous quantitative variables were calculated, and the baseline of continuous quantitative variables were evaluated by single factor analysis of variance. As for qualitative variables, the frequency, composition ratio and chi-square statistics were calculated under each category, and the chi-square test was used to evaluate the baseline of qualitative variables in different groups. Kaplan-Meier method was utilized to plot survival curves, and Cox proportional hazard model was applied to analyze the factors that might affect the prognosis for patients with stage II and III NSCLC. All tests were two-sided, and were performed by R 4.0.3. The abbreviation list was shown in Supplementary Table 1.

### Procedures

**Figure 1:**
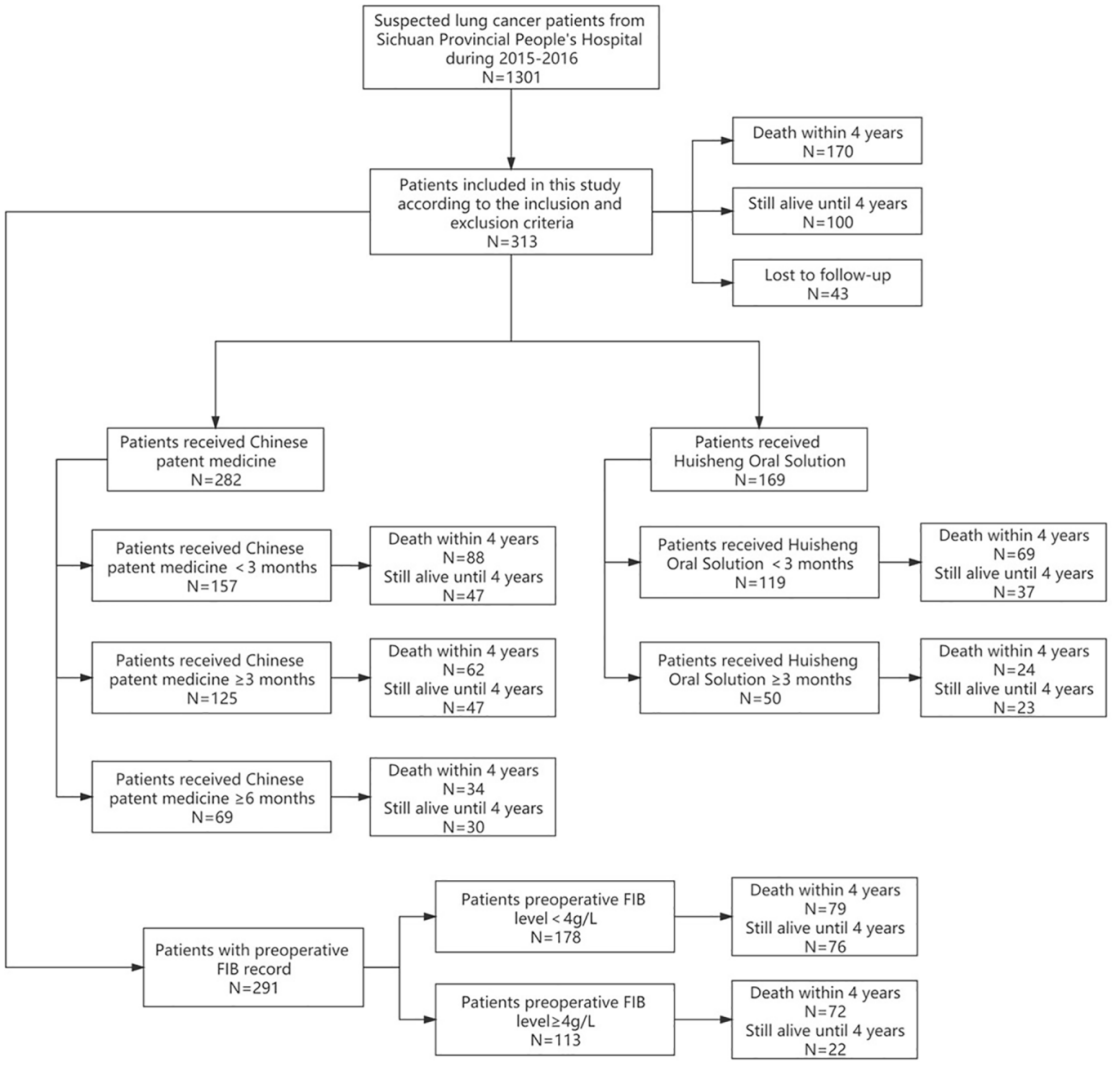
Research profile.

1301 patients were diagnosed with lung cancer in Sichuan Provincial People’s Hospital during 2015–2016, and 313 patients with stage II and III NSCLC were included in statistical analysis according to the inclusion and exclusion criteria (Supplementary Table 2). Firstly, the status of combination use of CPM and Western medicine was analyzed, including the frequency of using each treatment and classification analysis according to the characteristics of each CPM’s formula. Secondly, the differences of primary outcome between the patients who received CPM for less than 3 months, more than 3 months, and more than 6 months were compared, and the difference of primary outcome between the patients who received HSOS for less than 3 months and more than 3 months was also compared. Finally, Kaplan-Meier method and Cox proportional hazard model were applied to analyze the effect of other factors on patients’ outcomes. The follow-up period was 48 months (4 years). 43 patients lost to follow-up. The rate of lost to follow-up was 13.7% (Figure 1).

## RESULTS

### The summary of all treatment used in patients diagnosed with NSCLC in Sichuan Provincial People’s Hospital during 2015–2016

#### The frequency of each treatment used in 313 patients

**Figure 2:**
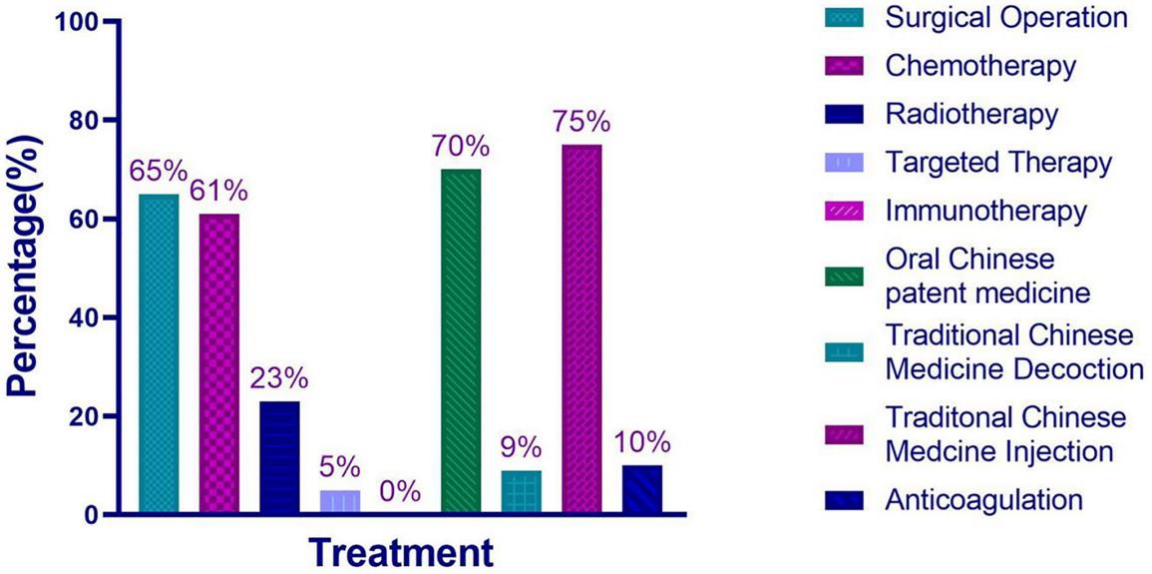
The frequency of each treatment used for 313 patients diagnosed with NSCLC in Sichuan Provincial People’s Hospital during 2015–2016.

The frequency of all kinds of treatments for patients diagnosed with stage II and III NSCLC in Sichuan Provincial People’s Hospital during 2015–2016 was shown in Figure 2. The frequency of surgery was 65%, chemotherapy was 61%, radiotherapy was 23%, targeted therapy was 5%, oral CPM was 70%, TCM decoction was 9%, CPM injection was 75%, and oral anticoagulant drug was 10%. None of patients received immunotherapy.

### Analysis of different types of Chinese patent medicine

In view of the fact that syndrome differentiation for patients was not the same, we conducted further analysis of different types of Chinese patent medicine received by patients with stage II and III NSCLC. A total of 8 kinds of Chinese patent medicine related to tumor treatment were prescribed in Sichuan Provincial People’s Hospital, and all of these Chinese patent medicines were classified into “Jiedu Kangai (解毒抗癌)” and “Fuzheng Quyu (扶正祛瘀)” according to the effect and characteristics of their formula. The results were listed as follows (Table 1).

**Table 1:** The treatment duration of two types of Chinese patent medicine for 313 patients (month).

**Type**	**Never used**	**Treatment duration <3 M**	**3 M ≤ Treatment duration <6 M**	**Treatment duration ≥6 M**	**Total**
**Jiedu kangai**					
Cinobufacin capsule	296 (94.6%)	7 (2.2%)	3 (1.0%)	7 (2.2%)	313
Fructus bruccae emulsion	134 (42.8%)	176 (56.2%)	3 (1.0%)	0 (0%)	313
**Fuzheng quyu**					
Huisheng oral solution	144 (46.0%)	119 (38.0%)	17 (5.4%)	33 (10.6%)	313
Fu-fangbanmao capsule	173 (55.3%)	129 (41.2%)	5 (1.6%)	6 (1.9%)	313
Kanglixin capsule	238 (76.1%)	52 (16.6%)	11 (3.5%)	12 (3.8%)	313
Aidi injection	179 (57.2%)	134 (42.8%)	0 (0%)	0 (0%)	313

### Comparison of baseline characteristics among patients who received CPM/HSOS for different durations (less than 3 months vs. more than 3 months vs. more than 6 months)

#### Baseline characteristics among patients who received CPM for different durations (less than 3 months vs. more than 3 months vs. more than 6 months)

There was no statistical significance between the baseline characteristics of three groups. The patients between three groups were comparable. Results were listed as follows (Table 2).

**Table 2:** Comparison of baseline characteristics between 3 groups with different treatment duration of Chinese patent medicine for 313 patients.

**Variable**	**Treatment duration <3 M**	**Treatment duration ≥3 M**	**Treatment duration ≥6 M**	**Statistics**	***P*-value**
**Gender**					
Male	132	100	52	χ^2^ = 3.816	*P* = 0.148
Female	56	25	17		
**Age**	63.22 (±9.85)	62.89 (±9.96)	63.59 (±10.30)	*F* = 0.115	*P* = 0.892
**Stage**					
II	72	46	25	χ^2^ = 0.124	*P* = 0.940
III	116	79	44		
**Pathological types**					
Squamous carcinoma	62	50	22	Fisher’s exact test	*P* = 0.214
Adenocarcinoma	83	59	38		
Poor differentiated carcinoma	9	5	3		
Large cell carcinoma	0	1	0		
Spindle cell carcinoma	1	0	0		
Other types	33	10	6		
**Blood routine**					
PLT	209.34 (±83.02)	211.71 (±86.22)	205.79 (±94.2)	*F* = 0.102	*P* = 0.813
**Coagulation indicators**					
FIB	3.90 (±1.42)	3.80 (±1.26)	3.82 (±1.33)	*F* = 0.207	*P* = 0.989
D-Dimer	0.92 (±2.31)	1.22 (±4.16)	2.13 (±5.96)	*F* = 0.581	*P* = 0.561
TT	18.26 (±2.20)	18.27 (±1.96)	18.17 (±1.83)	*F* = 0.059	*P* = 0.942
PT	11.40 (±0.99)	11.21 (±0.84)	11.23 (±0.89)	*F* = 1.031	*P* = 0.359
APTT	28.20 (±4.23)	28.33 (±4.70)	28.15 (±4.35)	*F* = 0.027	*P* = 0.974

#### Baseline characteristics between patients who received HSOS for less than 3 months and patients who received HSOS for more than 3 months

There was no statistical significance in the difference of baseline characteristics, which indicated that the patients were comparable between two groups. The results were listed as follows (Table 3).

**Table 3:** Comparison of baseline characteristics between 2 groups with different treatment duration of Huisheng oral solution for 169 patients.

**Variable**	**Treatment duration <3 M**	**Treatment duration ≥3 M**	**Statistics**	***P*-value**
**Gender**				
Male	91	36	χ^2^ = 0.642	*P* = 0.423
Female	26	14		
**Age**	62.28 (±10.11)	63.88 (±9.62)	*t* = −0.949	*P* = 0.344
**Stage**				
II	47	17	χ^2^ = 0.564	*P* = 0.453
III	70	33		
**Pathological types**				
Squamous carcinoma	49	16	Fisher’s exact test	*P* = 0.583
Adenocarcinoma	51	27		
Poor differentiated carcinoma	3	3		
Large cell carcinoma	1	0		
Spindle cell carcinoma	1	0		
Other types	12	4		
**Blood routine**				
PLT	216.44 (±80.84)	200.80 (±104.00)	*t* = 1.034	*P* = 0.302
**Coagulation indicators**				
FIB	3.88 (±1.34)	3.72 (±1.33)	*t* = 0.696	*P* = 0.488
D-Dimer	0.58 (±0.65)	2.78 (±7.46)	*t* = −1.473	*P* = 0.151
TT	18.43 (±1.98)	18.11 (±1.87)	*t* = 0.953	*P* = 0.342
PT	11.32 (±0.98)	11.17 (±0.74)	*t* = 0.740	*P* = 0.461
APTT	27.81 (±3.94)	28.59 (±4.70)	*t* = −0.834	*P* = 0.407

### 4-year survival rate for patients treated with different durations of CPM/HSOS

#### Survival analysis of patients who received CPM for less than 3 months and patients who received CPM for more than 3 months

**Figure 3:**
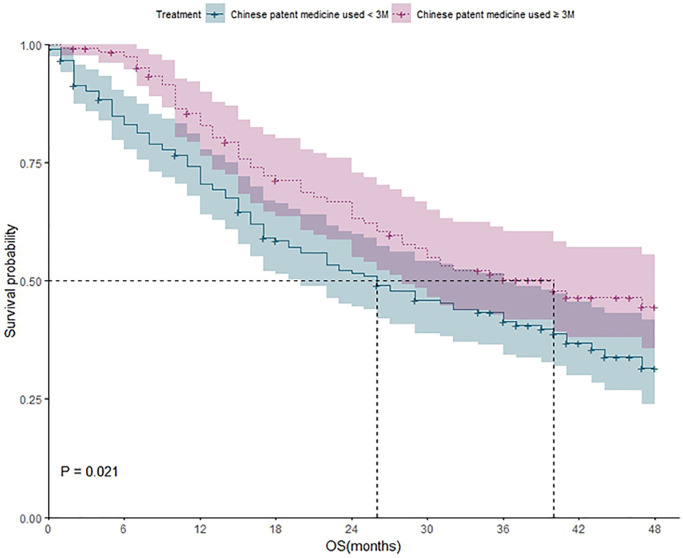
The survival analysis of patients who received CPM for different durations (less than 3 months vs. more than 3 months).

**Figure 4:**
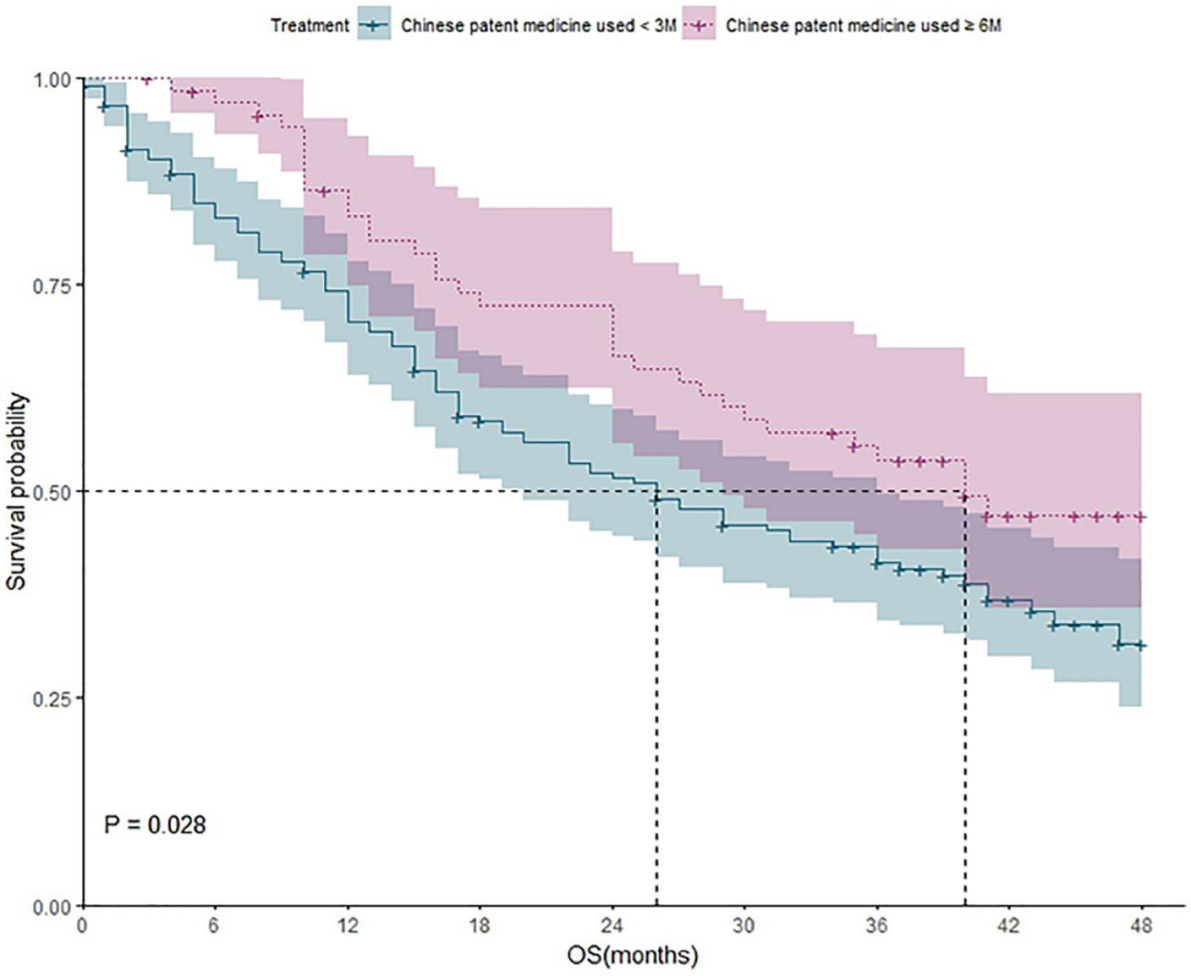
The survival analysis of patients who received CPM for different durations (less than 3 months vs. more than 6 months).

**Figure 5:**
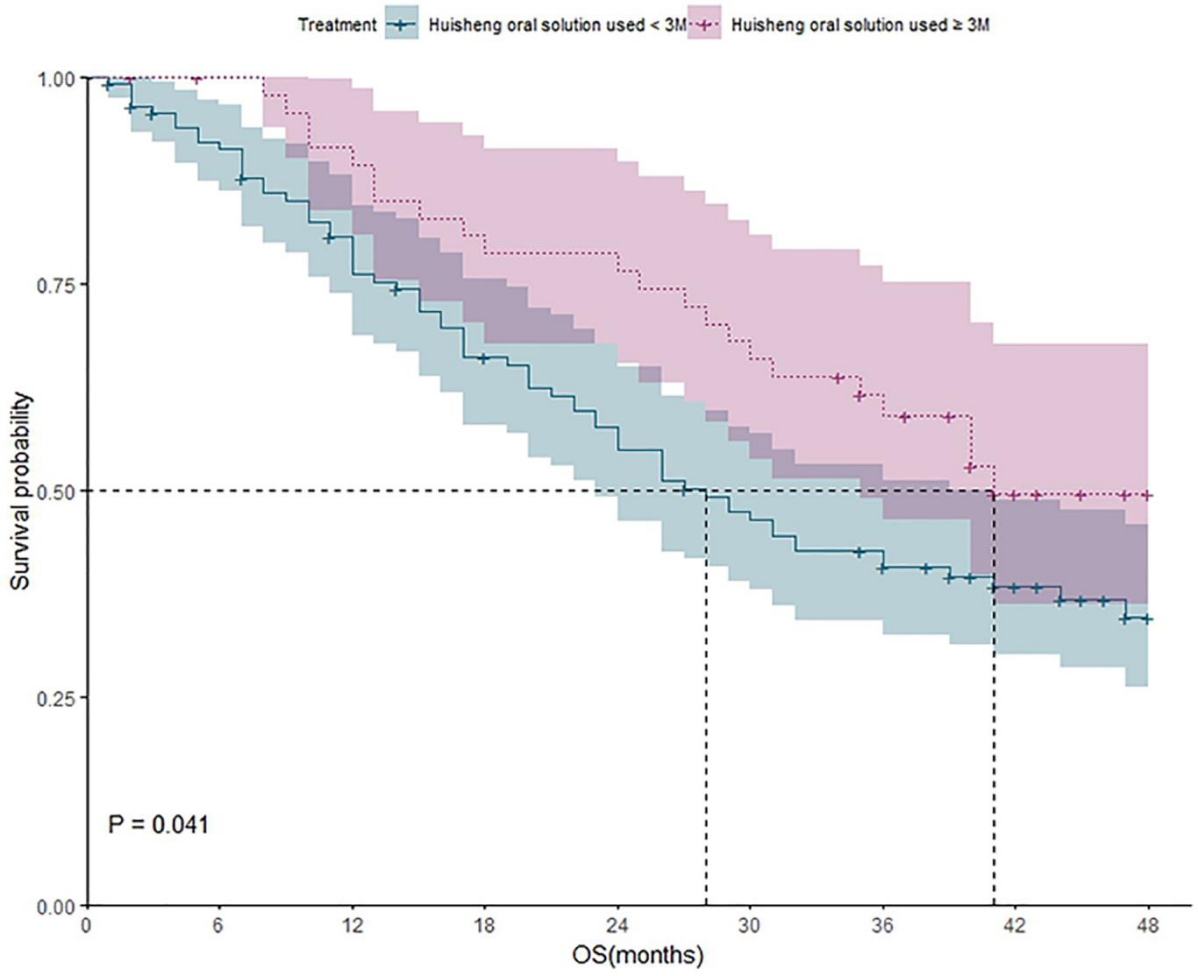
The survival analysis of patients who received HSOS for different durations (less than 3 months vs. more than 3 months).

188 patients received CPM less than 3 months, 105 of them died during the study. The one-year, two-year, three-year, and four-year survival rate were 70%, 51%, 41%, and 31%, respectively. 125 patients received CPM for more than 3 months, and 62 of them died during the study. The one-year; two-year; three-year and four-year survival rate were 83%, 63%, 50%, and 42%, respectively. Kaplan-Meier method was applied to analyze the difference of outcomes between two groups, and the results showed that the difference between two groups was statistically significant (*P* = 0.021), which indicated that the 4-year survival rate of patients with CPM used for ≥3 months was higher than that of patients with CPM used for <3 months (Figure 3). The shadow of two survival curves in different color represented the 95% confidence interval of survival probability for each curve, which also applied to Figures 4 and 5.

#### Survival analysis of patients who received CPM for less than 3 months and patients who received CPM for more than 6 months

188 patients received CPM for less than 3 months, and 105 of them died during the study. The one-year, two-year three-year, and four-year survival rate were 70%; 51%; 41%, and 31%, respectively. 69 patients received CPM for more than 6 months, and 34 of them died during the study. The one-year, two-year, three-year, and four-year survival rate were 83%, 66%, 53%, and 43% respectively. Kaplan-Meier method was applied to analyze the difference of outcomes between two groups, and the result showed that the difference between the two groups was statistically significant (*P* = 0.028). It revealed that the 4-year survival rate of patients with CPM used for ≥6 months was higher than that of patients with CPM used for <3 months (Figure 4).

#### Survival analysis of patients who received HSOS less than 3 months and patients who received HSOS more than 3 months

117 patients received HSOS less than 3 months, and 68 of them died during the study. The one-year, two-year, three-year, and four-year survival rate were 75%, 54%, 40%, and 35%, respectively. 50 patients received HSOS more than 3 months, and 22 of whom died during the study. The one-year, two-year, three-year, and four-year survival rate were 89%, 77%, 60%, and 51%, respectively. Kaplan-Meier method was applied to analyze the difference of outcomes between two groups, and the result showed that the difference between two groups was statistically significant (*P* = 0.041), which demonstrated that the 4-year survival rate of patients with HSOS used for ≥3 months was higher than that of patients with HSOS used for <3 months (Figure 5).

### Multivariate analysis via Cox proportional hazard model

**Figure 6:**
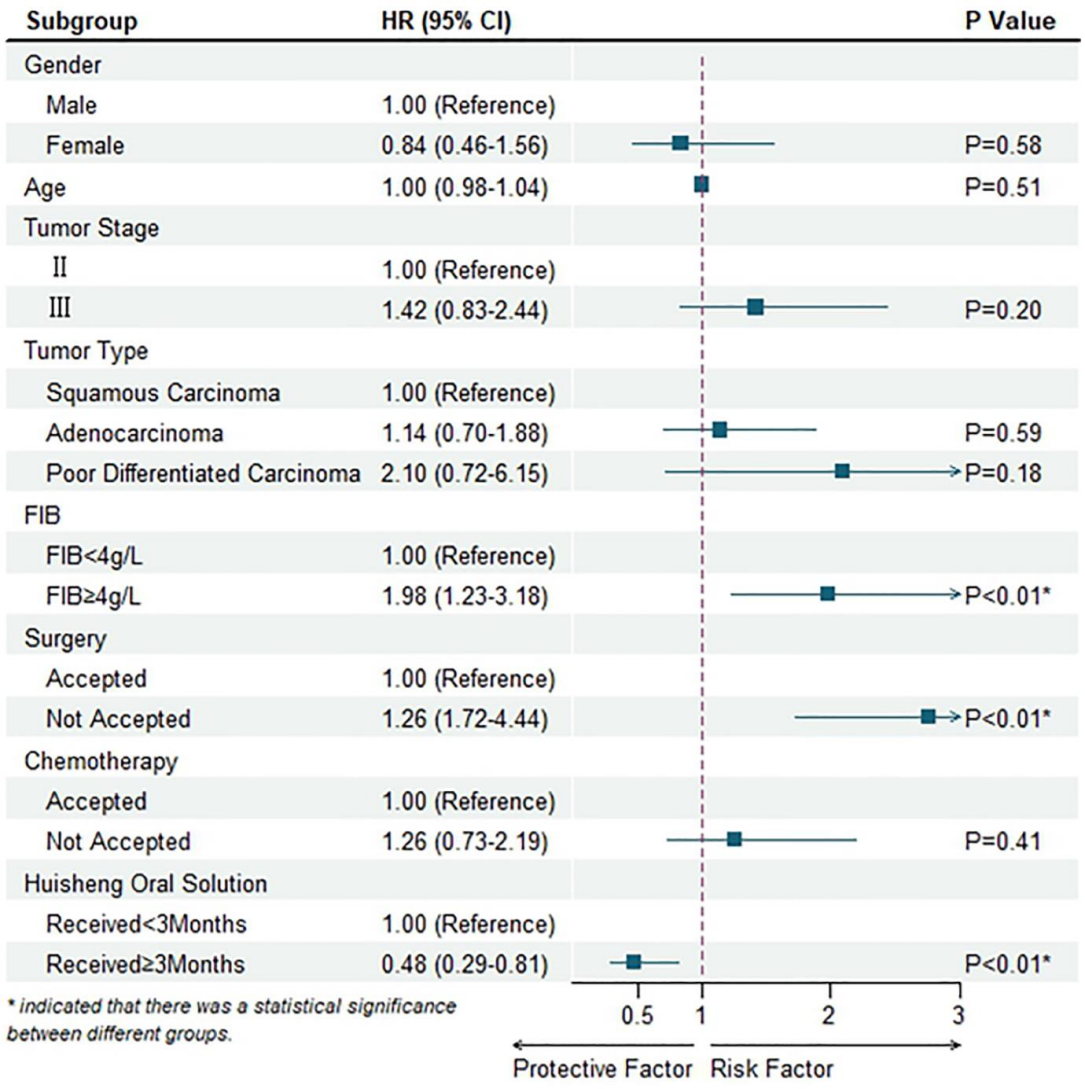
Multivariable Cox regression mode.

The factors that might affect the survival outcome of patients were included in the Cox proportional hazard model. The results indicated that elevated preoperative FIB level (*P* < 0.01), surgery (*P* < 0.01), and patients received HSOS more than 3 months were statistically significant in the model (*P* < 0.01). The hazard of death for patients with preoperative FIB level more than 4 g/L was 1.98 times higher than that of patients with preoperative FIB level less than 4 g/L, and for patients without surgery was 2.76 times higher than that of patients underwent surgery. Besides, we also found that the hazard of death for patients who received HSOS more than 3 months was just half that of patients who received HSOS less than 3 months (Figure 6 and Table 4).

**Table 4:** Multivariable Cox regression model.

**Variable**	**Hazard ratio (95% CI)**	***P*-value**
**Gender**		
Male	1.00 (Reference)	*P* = 0.58
Female	0.84 (0.46–1.56)	
**Age**	1.00 (0.98–1.04)	*P* = 0.51
**Tumor stage**		
II	1.00 (Reference)	*P* = 0.20
III	1.42 (0.83–2.44)	
**Tumor type**		
Squamous carcinoma	1.00 (Reference)	
Adenocarcinoma	1.14 (0.70–1.88)	*P* = 0.59
Poor differentiated carcinoma	2.10 (0.72–6.15)	*P* = 0.18
**FIB**		
FIB <4 g/L	1.00 (Reference)	*P* < 0.01^*^
FIB ≥4 g/L	1.98 (1.23–3.18)	
**Surgery**		
Accepted	1.00 (Reference)	*P* < 0.01^*^
Not accepted	2.76 (1.72–4.44)	
**Chemotherapy**		
Accepted	1.00 (Reference)	*P* = 0.41
Not accepted	1.26 (0.73–2.19)	
**Huisheng oral solution**		
Received <3 months	1.00 (Reference)	*P* < 0.01^*^
Received ≥3 months	0.48 (0.29–0.81)	

^*^Indicated that there was a statistical significance between different groups.

### Safety assessment

No adverse reactions related to HSOS were recorded in this study. All of the adverse reactions documented in the included medical records were evaluated as side effect of radiotherapy and chemotherapy by doctors in this research. HSOS has been prescribed for clinical use more than 20 years. A total of 46 cases of adverse reactions were found in the database of National Adverse Drug Reaction Monitoring System, which were all new and general adverse reactions [23, 24]. The national adverse drug reaction information and pharmacovigilance notice related to HSOS were not received, and no serious adverse reactions had been reported from clinical researches (Table 5).

**Table 5:** The summary of adverse reactions for HSOS from national adverse drug reaction monitoring system.

**No.**	**Adverse reaction**	**Occurred time**	**Prognosis**	**The reason of using HSOS**	**Remark**
1	Diarrhea	2011-11-17	Improved	Lung cancer treatment	General adverse reaction
2	Fatigue, myalgia	2011-3-26	Improved	Esophageal cancer treatment	General adverse reaction
3	Dyspnea, chest tightness, insomnia	2012-3-5	Improved	Liver cancer treatment	General adverse reaction
4	Nausea, emsis	2012-3-3	Improved	Laryngopharynx cancer treatment	General adverse reaction
5	Fever	2012-4-5	Recovery	Cancer treatment	General adverse reaction
6	Nausea, diarrhea	2012-11-1	Recovery	Cancer treatment	General adverse reaction
7	Emesis	2014-8-22	Recovery	Eliminating disease, and removing blood stasis	General adverse reaction
8	Abdominalgia	2014-9-4	Recovery	Eliminating disease, and removing blood stasis	General adverse reaction
9	Rash, pruritus	2014-7-20	Recovery	Eliminating disease, and removing blood stasis	General adverse reaction
10	Emesis, nausea	2014-7-16	Improved	Lung cancer treatment	General adverse reaction
11	Waist soreness	2014-7-28	Unknown	Adjuvant therapy for cancer treatment	General adverse reaction
12	Dizziness	2014-7-22	Improved	Cancer treatment	General adverse reaction
13	Nausea, abdominalgia	2014-10-10	Improved	Cancer treatment	General adverse reaction
14	Rash, pruritus	2014-9-21	Recovery	Lung cancer treatment	General adverse reaction
15	Nausea, emesis	2014-11-26	Improved	Cancer treatment	General adverse reaction
16	Ulcer	2014-8-6	Recovery	Eliminating disease, and removing blood stasis	General adverse reaction
17	Rash	2015-6-26	Improved	Combined with other chemotherapy	General adverse reaction
18	Nausea, emesis	2015-11-6	Improved	Cancer treatment	General adverse reaction
19	Oral ulcer	2015-9-18	Recovery	Trachea, bronchus and lung cancer treatment	General adverse reaction
20	Nausea	2015-4-20	Improved	Gastric cancer treatment	General adverse reaction
21	Emesis	2015-5-10	Improved	Esophageal cancer treatment	General adverse reaction
22	Rash	2015-11-26	Recovery	Symptomatic treatment	General adverse reaction
23	Nausea, emesis	2016-5-3	Improved	Adjuvant therapy	General adverse reaction
24	Nausea	2016-6-6	Improved	Pneumonia treatment	General adverse reaction
25	Nausea	2016-9-7	Improved	Breast cancer treatment	General adverse reaction
26	Nausea	2016-9-16	Improved	Fibrosarcoma treatment	General adverse reaction
27	Diarrhea, abdominalgia	2016-5-27	Improved	Lung cancer	General adverse reaction
28	Severe pain	2016-10-6	Improved	Eliminating disease, and removing blood stasis	General adverse reaction
29	Dizziness, emesis	2016-11-22	Improved	Lung cancer treatment	General adverse reaction
30	Abdominal distension	2016-11-27	Improved	Cancer treatment	General adverse reaction
31	Dermatologic diseases	2017-2-23	Maintain	Cancer treatment	General adverse reaction
32	Diarrhea, abdominalgia, nausea, emesis	2017-2-8	Improved	Breast cancer treatment	General adverse reaction
33	Pruritus	2017-12-3	Improved	Cancer treatment	General adverse reaction
34	Diarrhea, dizziness	2018-4-29	Improved	Gastritis, bronchitis and lung cancer treatment	General adverse reaction
35	Papule, pruritus	2018-8-7	Improved	Lung cancer treatment	General adverse reaction
36	Rash	2018-9-28	Improved	Cancer treatment	General adverse reaction
37	Erythematous eruption	2018-12-16	Improved	Lung cancer treatment	General adverse reaction
38	Chill, Cyanosis	2019-7-22	Improved	Cancer treatment	General adverse reaction
39	Emesis,	2019-12-25	Improved	Cancer treatment	General adverse reaction
40	Rash, pruritus	2020-5-8	Improved	Cancer treatment	General adverse reaction
41	Skin allergy	2020-6-25	Recovery	Eliminating disease, and removing blood stasis	General adverse reaction
42	Eilema	2020-8-13	Recovery	Gastric cancer treatment	General adverse reaction
43	Dizziness, instability of gait	2020-10-28	Recovery	Lung cancer treatment	General adverse reaction
44	Abdominalgia	2020-6-15	Improved	Adjuvant therapy for cancer treatment	General adverse reaction
45	Nausea	2021-1-3	Recovery	Strengthen body	General adverse reaction
46	Diarrhea	2021-1-22	Improved	Rheumatoid arthritis treatment	General adverse reaction

### The names and traditional use of botanical compositions in HSOS

The botanical compositions and traditional use or pharmacological effect of HSOS were listed as follows (Table 6).

**Table 6:** The names and traditional use of botanical composition in Huisheng oral solution.

**No.**	**Accepted name**	**Chinese name**	**Latin name**	**Traditional use or pharmacological effect**
1	Leonurus japonicus Houtt.	益母草	Leonuri Herba	Reduction of whole blood viscosity and platelets aggression (Liu XH et al., 2007)
2	Sparganium stoloniferum (Buch.-Ham. ex Graebn.) Buch.-Ham. ex Juz.	三棱	Sparganii Rhizoma	Anti-tumor and antithrombotic effects (Jia J et al., 2020)
3	Dalbergia odorifera T.C.Chen.	降香	Lignum Dalbergiae Odoriferae	Common use for promoting blood circulation, relieving pain and removing blood stasis (Yang ZH et al., 2013)
4	Alpinia officinarum Hance.	高良姜	Alpiniae Officinarum Rhizoma	Application to treating inflammation, pain, and stomach-ache (Abubakar IB et al., 2018)
5	Prunus armeniaca L.	苦杏仁	Armeniacae Semen Amarum	Application to treating dyschesia, constipation, abdominal fullness, fatigue and pale tongue (Gao L et al., 2014)
6	Foeniculum vulgare Mill.	小茴香	Foeniculi Fructus	Wide use as an antiemetic ameliorating stomach conditions or an analgesic (Lee JH et al., 2012)
7	Corydalis yanhusuo (Y.H.Chou and Chun C.Hsu) W.T.Wang ex Z.Y.Su and C.Y.Wu	延胡索	Corydalis Rhizoma	Antithrombotic, antimicrobial and anti-inflammation activity (Tian B et al., 2020)
8	Boswellia carteri Birdw.	乳香	Olibanum	Anti-inflammatory, anti-arthritic and anti-cancer effects (Choi OB et al., 2009)
9	Ferula sinkiangensis K.M.Shen.	阿魏	Ferulae Resina	Use as an antioxidant with chemo-preventive properties in cancer (Bagheri SM et al., 2016)
10	Rehmannia glutinosa (Gaertn.) DC.	熟地黄	Rehmannia glutinosa Libosch	Anti-inflammatory, anti-microbial, and anti-tumor effects (Bhattamisra SK et al., 2019)
11	Carthamus tinctorius L.	红花	Carthami Flos	Treating blood stasis and activating blood (Guo YF et al., 2014)
12	Angelica sinensis (Oliv.) Diels.	当归	Angelicae Sinensis Radix	Use of treating anemiafor invigorating blood circulation and modulating the balance of the immune system (Liu J et al., 2019)
13	Anemone raddeana Regel.	两头尖	Anemones Raddeanae Rhizoma	Application to conditions such as wind and cold symptoms, hand-foot disease and spasms, and joint pain and ulcer pain (Wang SS et al., 2020)
14	Cyperus rotundus L.	香附	Cyperi Rhizoma	The effect of activating blood circulation and dissipating blood stasis (Liu P et al., 2017)
15	Curcuma longa L.	姜黄	Curcumae Longae Rhizoma	Application to relieving stagnation and stasis, alleviating pain, and curing amenorrhea and wounds (Chen Z et al., 2017)
16	Rheum palmatum L.	大黄	Rhei Radix et Rhizoma	Use of treating liver diseases, constipation and inflammation (Pan TL et al., 2015)
17	Prunus persica (L.) Batsch	桃仁	Persicae Semen	Activating blood, removing stasis and loosing bowels to relieve constipation (Xi S et al., 2013)
18	Paeonia lactiflora Pall.	白芍	Paeoniae Radix Alba	Wide use of treating blood diseases, night sweating, contracture of the limbs, and headache (Tan YQ et al., 2020)
19	Cinnamomum cassia (L.) J.Presl	肉桂	Cinnamomi Cassiae Cortex	Anti-platelet aggregation and antioxidative, anti-diabetic, and anti-fungal activities (Zhou W et al., 2018)
20	Zanthoxylum bungeanum Maxim.	花椒	ZanthoxμLi Pericarpium	Anti-platelet aggregation, anti-inflammatory activity, antioxidant, and anti-cancer activity (Diao WR et al., 2013)
21	Caesalpinia sappan L.	苏木	Sappan Lignum	Promoting blood circulation, removing blood stasis, alleviating pain, and anti-tumor activity (Yang X et al., 2016)
22	Ligusticum chuanxiong Hort.	川芎	Chuanxiong Rhizoma	Anti-thrombotic, anti-inflammatory, anti-cancer, and anti-oxidant effects (Chen Z et al., 2018)
23	Panax ginseng C.A.Mey.	人参	Ginseng Radix et Rhizoma	Application to enhancing body immunity and preventing cancer (Yu S et al., 2020)
24	Commiphora myrrha (Nees) Engl.	没药	Myrrha	Antioxidant and anti-inflammatoryactivity (Ahmad A et al., 2015)
25	Perilla frutescens (L.) Britton	紫苏子	Perillae Fructus	Use of treating respiratory diseases, such as coughing and wheezing (Yim YK et al., 2010)
26	Syzygium aromaticum (L.) Merr. and L.M.Perry	丁香	Caryophylli Flos	Anti-microbial activity (Wong RW et al., 2010) Application to treating backache (Sun M et al., 2019)
27	Typha angustifolia L.	蒲黄	Typhae Pollen	Use of promoting blood circulation and removing stasis (Zeng H et al., 2016)
28	Euodia ruticarpa (A. Juss.) Benth.	吴茱萸	Evodiae Fructus	Analgesic, anti-emetic, anti-inflammatory and antidiarrheal effects (Cai Q et al., 2014)
29	Artemisia argyi H.Lév. and Vaniot	艾叶	Artemisiae Argyi Folium	Antibacterial, antiviral, anti-tumor and anti-inflammatory effects in relieving cough and asthma (Xiao-Yan L et al., 2020)

## DISCUSSION

According to the results of this retrospective study and relevant literature, we made a preliminary hypothesis on the mechanism that HSOS could benefit patients with tumor by improving blood hypercoagulable state and tumor micro-environment of patients. However, there were many different theories about the mechanism of tumor recurrence and metastasis in TCM and Western Medicine. The theory of TCM held that recurrence and metastasis of the tumor were related to “Yuxie” and “Fudu”. The mechanism of the tumor recurrence was closely correlated to the existence of “Yuxie” and the weakness of “Zhengqi”, and the remaining tumor cells after surgery could become “Duxie” hidden in the body, waiting to be induced [15]. Thus, the main manifestation of this situation was recurrence of the tumor, which was difficulty to cure. The “seed and soil” theory held that recurrence and metastasis of the tumor was the result of interaction between tumor cells and their micro-environment [25]. The micro-environment where the tumor located was complicated, which included various types of cells and cytokines [26, 27]. Some of these cytokines could further induce the expression of vascular endothelial growth factor (VEGF), promote tumor angiogenesis, and eventually lead to tumor recurrence and metastasis.

HSOS was an anti-tumor CPM with the effect of “eliminating disease”, “removing blood stasis”, and “strengthening body”. The previous study *in vitro* has shown that it could inhibit the proliferation of lung cancer cells and reduce the positive expression of CyclinD1 in lung adenocarcinoma cells. Some relevant experiments have also demonstrated that HSOS could improve tumor micro-environment and hypercoagulable state. Liu SQ and Wang W found that the FIB and D-dimer levels of SD rats treated with HSOS decreased significantly after 72 hours [19, 20]. Chen Z showed that the tumor micro-environment was considerably improved with the concentration of FIB, tissue factor, VEGF, and IL-6 decline after administration of HSOS [28]. Besides the effect on lung cancer, HSOS was also demonstrated that it could exert therapeutic effect on other types of tumor, such as ovarian, liver and esophageal cancer. Patients with different types of cancer mentioned above received HSOS combined with convention treatment (such as chemotherapy and radiotherapy) showed better outcomes than those who received convention treatment merely [29]. In this retrospective study, we found that survival benefits were highly associated with medium to long-term (more than 3 months) treatment of CPM/HSOS. The median survival time of patients who received HSOS intervention more than 3 months was longer than that of patients who received HSOS less than 3 months. Patients with elevated preoperative FIB level (more than 4 g/L) and those did not undergo surgery had a higher probability of cancer death, which was consistent with our previous study [30, 31]. Our previous study revealed that more than 3 months treatment of CPM/HSOS could improve 2-year survival rate of patients with stage II–III NSCLC, and preoperative FIB level more than 4 g/L was a risk factor for the prognosis [30]. The results did not change as follow-up time extended from 2 years to 4 years, and the hazard of death for patients with FIB more than 4 g/L was nearly 2 times than those with FIB less than 4 g/L, which to some extent strengthened the reliability of our study. Besides, we conducted further analysis involving patients with FIB ≥4 g/L, which could be regarded as a case that a patient was in hypercoagulable state, to compare outcomes for those who received HSOS more than 3 months and less than 3 months. However, the results showed that the difference of overall 4-year survival rate between FIB ≥4 g/L patients who received HSOS more than 3 months and less than 3 months was not statistically significant (*P* = 0.29), the median survival was longer for patients in hypercoagulable state and received HSOS more than 3 months compared to the contrast (31 months vs. 19 months), which to some extent demonstrated that HSOS could improve hypercoagulable state to prolong overall survival. There were also several shortcomings in our research: Firstly, this retrospective study was based on medical records from Hospital Information System and telephone visit, therefore, data missing and confounding bias were inevitable. Secondly, owing to the nature of retrospective design, it was difficult for us to verify the actual medical condition of each patient or to figure out the reasons why patients dropout, which might affect the results of the study. Thirdly, since many different types of medicine simultaneously or successively prescribed to the patients in actual treatment process, we could not distinguish the effect of each CPM in the combined treatment, especially in the case of short cumulative treatment duration (less than 3 months, Cumulative treatment duration ≥3 months was set as the effective exposure dose for our survival analysis [14, 15], but only to make a preliminary assessment on its curative effect. The missing value of coagulation indicators after the long-term intervention of HSOS also impeded our exploration on their relationship [32, 33]. Therefore, a prospective clinical research would be anticipated to investigate whether patients could benefit from adequate CPM intervention in the future.

Finally, due to the complex compositions of TCM, it was challenging for the existing science and technology to clarify the mechanism and mutual relationships between different Chinese medicine components. Therefore, it is better to take the TCM formula that has been widely used in clinical practice for verification, and then infer the mechanism after obtaining reliable and scientific data to develop its application in other disease fields instead of analyzing mechanism directly.

## Supplementary Materials

Supplementary Table 1

Supplementary Table 2
