# Supplementary material for: A retrospective study on the effect of Chinese patent medicine combined with conventional treatment on the survival outcomes of 313 patients with stage II–III NSCLC
Source: Aging (Albany NY). 2024 Mar 28;16(7):6212–28. doi: 10.18632/aging.205697 (PMC11042965; doi:10.18632/aging.205697)
Supplement: Supplementary Table 1 [file aging-16-205697-s001.pdf]

SUPPLEMENTARY TABLE

Supplementary Table 1. The abbreviation list.

Abbreviation	Full title
CPM	Chinese patent medicine
DFS	Disease free survival
FIB	Fibrinogen
HIS	Hospital information system
HSOS	Huisheng oral solution
NSCLC	Non-small cell lung cancer
OS	Overall survival
PLT	Platelet
TCM	Traditional Chinese medicine
